# Hsp104-Dependent Remodeling of Prion Complexes Mediates Protein-Only Inheritance 

**DOI:** 10.1371/journal.pbio.0050024

**Published:** 2007-01-23

**Authors:** Prasanna Satpute-Krishnan, Sara X Langseth, Tricia R Serio

**Affiliations:** Department of Molecular Biology, Cell Biology and Biochemistry, Brown University, Providence, Rhode Island, United States of America; University of California San Francisco, United States of America

## Abstract

Inheritance of phenotypic traits depends on two key events: replication of the determinant of that trait and partitioning of these copies between mother and daughter cells. Although these processes are well understood for nucleic acid–based genes, the mechanisms by which protein-only or prion-based genetic elements direct phenotypic inheritance are poorly understood. Here, we report a process crucial for inheritance of the Saccharomyces cerevisiae prion [*PSI^+^*], a self-replicating conformer of the Sup35 protein. By tightly controlling expression of a Sup35-GFP fusion, we directly observe remodeling of existing Sup35^[*PSI+*]^ complexes in vivo. This dynamic change in Sup35^[*PSI+*]^ is lost when the molecular chaperone Hsp104, a factor essential for propagation of all yeast prions, is functionally impaired. The loss of Sup35^[*PSI+*]^ remodeling by Hsp104 decreases the mobility of these complexes in the cytosol, creates a segregation bias that limits their transmission to daughter cells, and consequently diminishes the efficiency of conversion of newly made Sup35 to the prion form. Our observations resolve several seemingly conflicting reports on the mechanism of Hsp104 action and point to a single Hsp104-dependent event in prion propagation.

## Author Summary

The inheritance of phenotypic traits (the observable characteristics of the organism) is a fundamental process in biology. Most phenotypes are controlled by a cell's genes, and a particular phenotype becomes heritable when this underlying genetic information is copied and transmitted to progeny. In contrast, another group of phenotypes appears to be inherited through a protein-only, or prion, mechanism in which the structure of a protein rather than its sequence is the molecular determinant of the phenotype. It is thought that the presence of a prion in a cell forces conversion of a normal cellular protein into a differently folded shape (the prion form), which simultaneously deprives the cell of the protein's normal function and causes the prion-folded protein to aggregate within the cell. However, prion inheritance (how prions are passed down to daughter cells) remains poorly understood.

Using the yeast prion [*PSI^+^*] as a model system, we have elucidated a process necessary for protein-only inheritance. Here we show that the molecular chaperone Hsp104, a factor necessary for the inheritance of all known yeast prions, plays a single primary role in generating additional templates for protein-state replication. In the absence of this activity, existing prion templates are inefficiently transferred to daughter cells. As a consequence, the rate of protein-state replication is greatly decreased, and the protein-based phenotype is progressively lost.

## Abstract 3

The authors examine the role of the molecular chaperone Hsp104 in controlling inheritance of the prion form of Sup35^[*PSI+*]^.

## Introduction

The prion hypothesis was originally proposed to explain the etiology of the transmissible spongiform encephalopathies, a group of mammalian neurodegenerative diseases that arise and spread through a protein-only mechanism: a self-perpetuated change in the folding of a cellularly encoded protein PrP [1,2]. The idea that a protein conformation could encode genetic information gained additional support when two enigmatic traits in *Saccharomyces cerevisiae,* [*URE3*] and [*PSI^+^*], were identified as prions [3]. The [*URE3*] and [*PSI^+^*] phenotypes mimic loss-of-function mutations in the *URE2* and *SUP35* genes, respectively, but the prion phenotypes are epigenetic and arise when the Ure2 or Sup35 proteins assemble into functionally compromised higher-order complexes that are self-replicating [4–6]. Importantly, specific conformations of the Ure2 or Sup35 proteins confer heritable [*URE3*] and [*PSI^+^*] when exogenously supplied to non-prion cells, the ultimate proof of the prion mechanism for these traits [7–9].

The cornerstone of this protein-only mechanism is the ability of the prion form to self-replicate by templating the conversion of other conformers of the same protein to a like state. This process has been modeled in vitro for both Ure2 and Sup35, providing additional support for the prion hypothesis [10–13]. In each case, denatured recombinant protein spontaneously assembles into ordered, β sheet–rich fibers that bind amyloid-specific dyes upon dilution from denaturant. Remarkably, fibrillization of freshly added protein occurs at an accelerated rate in the presence of pre-formed fibers or of lysates from cells propagating the prion form, recapitulating the self-replication process.

**Figure 1:**
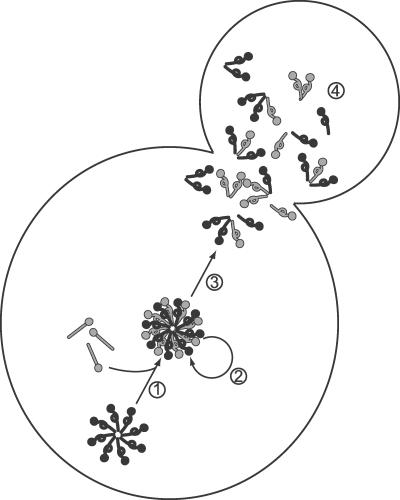
The In Vivo Prion Cycle Is a Multistep Pathway Existing prion complexes (black ball and loop) replicate by stimulating the conversion of either newly synthesized or non-prion conformer of the protein (gray ball and stick) to the prion form (black and gray ball and loop, step 1). Prion complexes must be stably maintained (step 2), but continually divided to generate new prion templates for additional rounds of protein-state replication (step 3). The smaller complexes generated by this division are efficiently transmitted to daughter cells (step 4).

Although prion proteins clearly have the capacity to direct the folding of other conformers in a defined purified system, extension of the prion hypothesis to practice becomes a multistep endeavor within the context of a living cell (Figure 1). First, newly synthesized prion protein must acquire the prion form, likely through its incorporation into existing prion complexes. Second, prion complexes must be stably maintained yet continually subdivided to yield additional replication templates known as prion seeds or propagons*.* Third, propagons must be partitioned to daughter cells in actively dividing cultures, where they continue to direct the conversion of newly made protein to the prion form and ensure inheritance of the associated phenotype. Thus, inhibition of the pathway at any point would lead to the same outcome: prion loss (also known as curing).

Progression through this in vivo prion cycle (Figure 1) is modulated by parallel, and sometimes competing, quality-control pathways [14], and the interplay between these processes has emerged as a powerful tool for dissecting the mechanism of protein-only inheritance in vivo. Not surprisingly, factors involved in the quality control of protein folding are the largest group of characterized prion regulators in yeast [15–22]. Members of both the Hsp70 (Ssa1 and Ssb1) and Hsp40 (Ydj1) families have been implicated in [*URE3*] [15–17] and [*PSI^+^*] [18,20–24] inheritance, but the molecular disaggregase Hsp104 has, by far, the most pronounced effects on fungal prion propagation.

Hsp104 is a member of the heat-inducible Clp/Hsp100 family of AAA^+^ ATPases and functions as a hexamer to resolve stress-induced aggregates [25–28]. Hsp104 is also constitutively expressed at low levels in logarithmically growing yeast cultures [28]. Under these normal growth conditions, Hsp104 is required for both the de novo appearance and propagation of all known S. cerevisiae prions [15,20,28–30]. Given its essential role, the mechanism by which Hsp104 mediates protein-only inheritance has been the subject of intense study.

In the case of [*PSI^+^*], deletion of Hsp104 induces prion loss or curing [20], and strains devoid of Hsp104 function cannot reacquire heritable [*PSI^+^*] [29], leading to the initial suggestion that Hsp104 activity was required for Sup35 to access and/or maintain a prion-competent intermediate form [5,20]. Contrary to this idea, [*PSI^+^*] can be induced de novo in the absence of Hsp104 if Sup35 is overexpressed along with a second prion protein such as New1 or Rnq1, suggesting that the Hsp104 requirement for de novo [*PSI^+^*] formation is actually an indirect effect through the propagation of a co-prion [30,31]. In line with this model, newly synthesized Sup35 can join existing complexes upon Hsp104 inhibition in vivo [32], and full-length Sup35, or a fragment of the protein comprising only the prion determining domain (NM), can assemble into amyloid fibers in the absence of Hsp104 in vitro [10,11]. However, Hsp104 does accelerate the spontaneous assembly of Sup35 fibers in vitro [13,33,34] and, at early time points in fibrillization reactions, promotes the steady-state accumulation of a Sup35 conformer that immunoreacts with an antibody specific for amyloidogenic intermediates [33]. Thus, although Hsp104 may not be absolutely required for conversion of Sup35 to the prion state, its activity may increase the efficiency of this process.

In addition to a potential role in protein-state replication, Hsp104 has been linked to the generation of additional [*PSI^+^*] propagons, a process most frequently described as fragmentation of prion complexes [6,35]. Such fragmentation would increase the converting capacity of the existing steady-state pool of Sup35 by distributing the protein among a greater number of complexes and would also facilitate the transmission of these prion templates to daughter cells upon division. In support of this model, prion loss upon inhibition of Hsp104 is progressive in dividing cultures, with preferential retention of propagons in mother cells [36,37]. Reactivation of Hsp104 in these cells prior to prion loss results in a cell division– and protein synthesis–independent increase in propagon number, suggesting an Hsp104-mediated change in existing Sup35 [32,37]. Although the biochemical nature of propagons is currently unknown, the steady-state size of Sup35 complexes is larger in cultures upon Hsp104 inhibition, consistent with a block to Hsp104-dependent fragmentation [38,39], and recent in vitro studies report the direct fragmentation of Sup35 or NM fibers by Hsp104 [33,34].

Despite these dynamic changes in Sup35 physical state during the prion cycle, existing prion complexes must remain sufficiently stable at steady state to maintain and transmit the prion phenotype. Recent reports, employing Sup35 or NM fusions to the green fluorescent protein (GFP) have suggested that Sup35 prion complexes are inherently unstable [40] and that Hsp104 is required to maintain the integrity of these complexes [41]. Consistent with this latter point, prion loss upon Hsp104 inhibition was reported in the absence of cell division, suggesting that curing occurs through the disassembly of existing prion oligomers [41]. However, these observations are at odds with previous reports. For example, resolubilized Sup35 is not biochemically detectible upon Hsp104 inhibition in [*PSI^+^*] cultures [32], and [*PSI^+^*] curing by loss of Hsp104 does not occur in non-dividing, stationary phase cultures [36,41].

Does Hsp104 actually mediate each step of the [*PSI^+^*] prion cycle in vivo, or does the interdependency of these events complicate interpretation of the available experimental observations? To resolve this issue, we have systematically probed each individual step in the [*PSI^+^*] prion cycle in vivo (Figure 1) by monitoring the Hsp104-dependent dynamics of either newly made or existing Sup35 in single live cells and correlating these changes in protein state to transitions in the prion phenotype. Our observations resolve many controversies in the literature and support a model in which Hsp104 acts at a single point in the [*PSI^+^*] prion cycle to remodel existing prion complexes. In the absence of this event, Sup35 complexes increase in size and decrease in mobility. As a result, these complexes are inefficiently transmitted to daughter cells, and newly synthesized Sup35 accumulates in a soluble form when the number of pre-existing oligomers becomes limiting.

## Results

### Sup35-GFP^[*psi−*]^ Efficiently Joins Existing Sup35^[*PSI+*]^ Complexes in the Absence of Hsp104 Function

**Figure 2:**
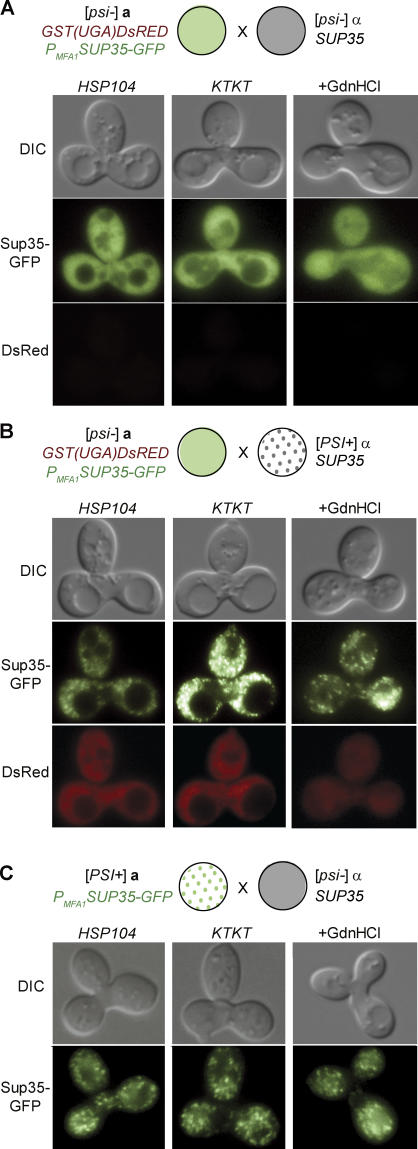
Efficient Incorporation of Sup35-GFP^[*psi−*]^ into Existing Sup35^[*PSI+*]^ Complexes Is Hsp104 Independent (A) *MAT**a*** [*psi^−^*] haploids expressing untagged *SUP35* from its endogenous locus, *SUP35-GFP* (green) from P*_MFA1_* and *GST(UGA)DsRED* from P*_GPD_* were mated to *MATα* [*psi^−^*] haploids constitutively expressing untagged *SUP35* (gray) in the presence or absence of functional Hsp104 as shown in the schematic diagram (top). The Sup35-GFP and DsRed fluorescence patterns were then examined in isolated zygotes. Since P*_MFA1_* is repressed upon mating [43], green fluorescence is derived from existing Sup35-GFP. Images of a representative zygote (*n* ≥ 5) from a wild-type cross (SY874 × 74D-694 *MATα;* left), from a cross in which the *MAT**a*** [*psi^−^*] mating partner contained a *KTKT* chromosomal replacement (SY876 × 74D-694 *MATα;* middle) or from a wild-type cross (SY874 × 74D-694 *MATα*) in the presence of GdnHCl (right) are shown. (B) *MAT**a*** [*psi^−^*] haploids constitutively expressing untagged *SUP35* and *GST(UGA)DsRED,* and *SUP35-GFP* (green) from P*_MFA1_* were mated to *MATα* [*PSI^+^*] haploids constitutively expressing untagged *SUP35* (gray dots) in the presence or absence of functional Hsp104 as shown in the diagram (top). Sup35-GFP and DsRed fluorescence were examined in isolated zygotes (*n* = 50). Images of representative zygotes from a wild-type cross (SY874 × 74D-694 *MATα;* left), from a cross in which the *MAT**a*** [*psi^−^*] mating partner contained a *KTKT* chromosomal replacement (SY876 × 74D-694 *MATα;* middle), or from a wild-type cross (SY874 × 74D-694 *MATα*) in the presence of GdnHCl (right) are shown. (C) *MAT**a*** [*PSI^+^*] P*_MFA1_ SUP35-GFP* haploids were mated to *MATα* [*psi^−^*] haploids constitutively expressing *SUP35* (gray) in the presence or absence of Hsp104 function, as indicated in the diagram (top). Images of a representative zygote (*n* ≥ 13) from a wild-type cross (SY597 × SY582; left), from a cross in which the *MATα* [*psi^−^*] mating partner contained a *KTKT* chromosomal replacement (SY597 × SY776 middle), or from a wild-type cross in the presence of GdnHCl (SY597 × SY582; right) are shown. The formation of zygotes was confirmed by sporulation (unpublished data).

[*PSI^+^*] is dominant in genetic crosses between [*PSI^+^*] and non-prion [*psi^−^*] haploids [42], and this dominance is established immediately upon cytoplasmic mixing through conversion of soluble Sup35 (Sup35^[*psi−*]^) to an oligomerized form (Sup35^[*PSI+*]^) [43]. We took advantage of this mating system to address the molecular mechanism by which Hsp104 mediates protein-only inheritance in vivo. For these experiments, we introduced Sup35-GFP^[*psi−*]^, a fully functional Sup35/GFP fusion [43], and untagged Sup35^[*PSI+*]^ into the same cell by mating. The fate of existing Sup35-GFP^[*psi−*]^ in the absence of new synthesis and in the presence or absence of Hsp104 function was then monitored in isolated zygotes (Figure 2). For these experiments, Hsp104 was selectively inactivated below the threshold of activity necessary for [*PSI^+^*] propagation in either of two ways. First, an Hsp104 variant, containing lysine-to-threonine substitutions in Hsp104′s two nucleotide-binding domains *(HSP104K218TK620T,* hereafter *KTKT),* was expressed as a dominant negative mutant [5,20,26]. Second, Hsp104′s ATPase activity was reversibly inhibited by guanidine hydrochloride (GdnHCl) [44–46].

In [*psi^−^*] cells, Sup35-GFP is evenly distributed throughout the cytoplasm in a diffuse pattern. Sup35-GFP^[*psi−*]^ retains this pattern upon mating to an unmarked [*psi^−^*] cell (Figure 2A), but coalesces into distinct cytoplasmic foci upon encountering Sup35^[*PSI+*]^ (Figure 2B) [43]. When similar matings are performed upon Hsp104 inhibition, diffuse Sup35-GFP^[*psi−*]^ fluorescence also coalesces into foci (Figure 2B). To rule out the possibility that Sup35-GFP^[*psi−*]^ changes form before Hsp104 inactivation, we photobleached existing Sup35-GFP in +/*KTKT* zygotes and monitored the fluorescence pattern of newly synthesized Sup35-GFP (Figure S1). Under these conditions, fluorescence again coalesces; thus, Sup35^[*psi−*]^ can join existing Sup35^[*PSI+*]^ complexes via an Hsp104-independent mechanism, an observation consistent with previous centrifugation studies [32].

Sup35 is the yeast homolog of eukaryotic release factor 3 [47], and Sup35^[*psi−*]^ incorporation into existing Sup35^[*PSI+*]^ complexes immediately and quantitatively inactivates the protein and induces a translation termination defect [43]. Since the persistence of minute quantities of soluble Sup35 in [*PSI^+^*] cells is sufficient to dominantly reverse the [*PSI^+^*] phenotype by supporting accurate translation termination [43], we followed accumulation of nonsense codon readthrough-dependent expression of a red fluorescent protein (DsRed) in zygotes as a measure of the efficiency of Sup35^[*psi−*]^ oligomerization in the presence or absence of Hsp104 (Figure 2). Although readthrough was not detected in [*psi^−^*] zygotes (Figure 2A), the [*PSI^+^*] phenotype was apparent in some [*PSI^+^*] × [*psi^−^*] zygotes independent of Hsp104 activity (Figure 2B). Given the sensitivity of this single-cell readthrough assay to soluble Sup35 [43], the appearance of red fluorescence in Hsp104-inactivated zygotes indicates the quantitative incorporation of Sup35^[*psi−*]^ into existing Sup35^[*PSI+*]^ complexes in these cells.

Although Sup35-GFP^[*PSI+*]^ foci are detected in all [*PSI^+^*] × [*psi^−^*] zygotes independent of Hsp104 activity, the percentage of these zygotes displaying the prion phenotype is affected by disaggregase function. In wild-type strains, only 80% of zygotes express DsRed within one cell division of mating, and this percentage is further decreased in Hsp104-inactivated zygotes (58% *KTKT,* 28% GdnHCl). Two previous studies provide possible explanations for this observation. First, GdnHCl has been shown to alter the efficiency of translation termination independent of [*PSI^+^*] [48]. Second, individual cells of a homogenous culture are known to fall within a broad range of propagon counts [37], and the penetrance of the [*PSI^+^*] phenotype in mating reactions likely reflects differences in converting capacity due to this range. We suggest that loss of Hsp104 exacerbates this cell-to-cell variation in converting capacity by blocking the generation of additional conversion templates (see below), as previously suggested [6,35,36].

### Existing Sup35^[*PSI+*]^ Complexes Become Immobile upon Hsp104 Inhibition

Although Sup35-GFP^[*psi−*]^ incorporation into existing Sup35^[*PSI+*]^ complexes is Hsp104-independent, we noted a difference in the appearance of Sup35-GFP foci in zygotes resulting from crosses between unmarked [*PSI^+^*] cells and [*psi^−^*] cells expressing Sup35-GFP in the presence or absence of Hsp104 function. In comparison with wild-type zygotes, Hsp104-inactivated zygotes contained brighter, more distinct foci (Figure 2B). This effect was prion dependent, as Hsp104 activity did not alter Sup35-GFP intensity in [*psi^−^*] × [*psi^−^*] zygotes (Figure 2A). However, this Hsp104-dependent distinction in the appearance of Sup35-GFP foci was lost when the fusion protein was expressed in the [*PSI^+^*] rather than the [*psi^−^*] mating partner (Figure 2C). These observations are consistent with a model in which the Hsp104-independent incorporation of Sup35-GFP^[*psi−*]^ into Sup35^[*PSI+*]^ complexes is rapidly followed by an Hsp104-dependent change in these oligomers, a process more easily visualized following the addition of a bolus of fluorescently marked Sup35 (Figure 2B).

**Figure 3:**
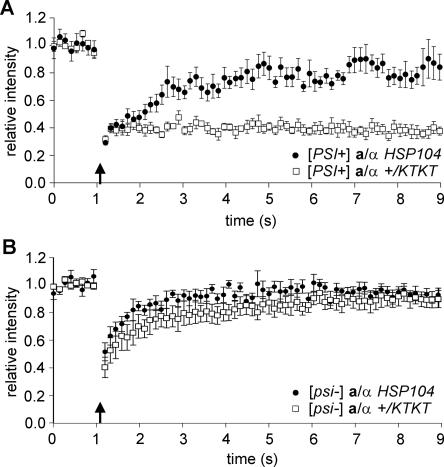
Hsp104 Inhibition Renders Sup35-GFP^[*PSI+*]^ Complexes Immobile (A) FRAP time courses for Sup35-GFP^[*PSI+*]^ in wild-type (SY360 × SY581; black circles) and in *+*/*KTKT* (SY893 × SY581; white squares) zygotes are shown (*n* = 6). The recovery half-time for the wild-type zygote is 0.92 s (R^2^ = 0.85). (B) FRAP time courses for Sup35-GFP^[*psi−*]^ in wild-type (SY360 × SY582; black circles, *n* = 5) and in *+*/*KTKT* (SY893 × SY582; white squares, *n* = 6) zygotes are shown. The recovery half-time for the wild-type zygote is 0.47 s (R^2^ = 0.85) and for the *+/KTKT* zygote is 0.7 s (R^2^ = 0.89). The black arrow marks the bleach point. Error bars represent standard error of the mean.

To directly test the idea that Sup35^[*PSI+*]^ complexes become altered upon Hsp104 inactivation, we probed the physical state of existing Sup35-GFP^[*PSI+*]^ with fluorescence recovery after photobleaching (FRAP) experiments. In wild-type zygotes, Sup35-GFP^[*PSI+*]^ was highly mobile, with 50% of the fluorescence recovered within 1 s (Figure 3A). Upon Hsp104 inactivation by *KTKT* (Figure 3A) or by GdnHCl treatment (Figure S2), Sup35-GFP^[*PSI+*]^ foci became largely immobile, with no recovery detected by 8 s. In contrast, Sup35-GFP^[*psi−*]^ recovery was rapid and Hsp104 independent (Figure 3B), indicating that Hsp104 specifically altered the biophysical properties of Sup35-GFP^[*PSI+*]^.

### Hsp104 Remodels Existing Sup35-GFP^[*PSI+*]^ Complexes

**Figure 4:**
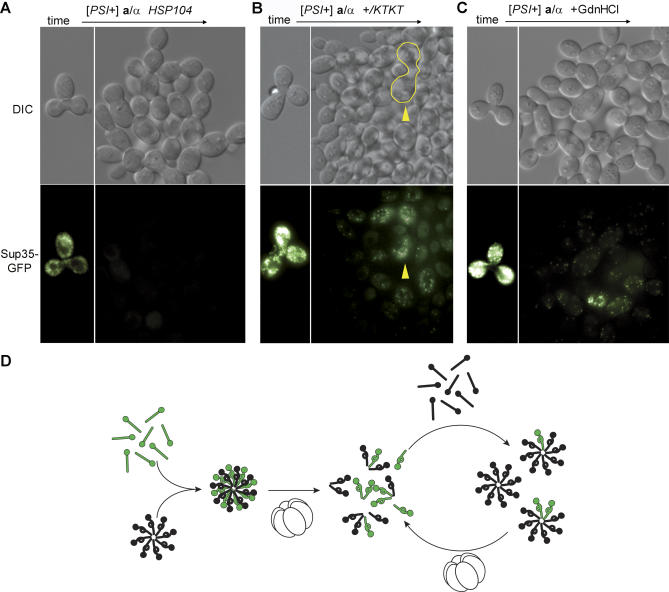
Hsp104 Inhibition Blocks Sup35-GFP^[*PSI+*]^ Remodeling and Creates a Segregation Bias for Sup35-GFP Complexes *MAT**a*** [*psi^−^*] haploids expressing untagged *SUP35* and *SUP35-GFP* from P*_MFA1_* were mated to *MATα* [*PSI^+^*] haploids constitutively expressing untagged *SUP35* in the presence or absence of functional Hsp104. For each cross, a zygote (left) and a microcolony derived from that zygote (right) are shown. (A) Representative images from a wild-type cross (SY360 × SY581) are shown (*n* = 10). (B) Shown are representative images (*n* = 5) from a *KTKT* × wild-type cross (SY876 × SY581). The originating zygote is outlined in the DIC image and marked with an arrow in the microcolony. (C) Shown are representative images (*n* = 6) of a wild-type cross (SY360 × SY581) in the presence of GdnHCl. (D) A model for Hsp104-dependent remodeling of prion complexes. Soluble Sup35-GFP^[*psi−*]^ (green ball and stick) is remodeled to the prion form (black and green ball and loop) upon encountering Sup35^[*PSI+*]^ in an Hsp104-independent process. These complexes are rapidly fragmented by Hsp104 (white hexamer) to smaller complexes, which then efficiently incorporate soluble Sup35^[*psi−*]^ (black ball and stick), effectively redistributing existing Sup35-GFP^[*PSI+*]^.

Given the change in Sup35-GFP^[*PSI+*]^ mobility upon inhibition of Hsp104, we reasoned that the dynamics of existing particles could be altered in these cells. To test this idea, we continued to monitor existing Sup35-GFP^[*PSI+*]^ as the zygotes formed microcolonies. In wild-type [*PSI^+^*] × [*psi^−^*] crosses, Sup35-GFP^[*PSI+*]^ foci became undetectable in small microcolonies (Figure 4A), as previously reported for the single fluorescent focus formed by a highly expressed NM-GFP fusion [40]. In contrast, Sup35-GFP^[*PSI+*]^ foci remained visible in Hsp104-inactivated [*PSI^+^*] × [*psi^−^*] microcolonies (Figure 4B and 4C). This persistence of existing complexes was not simply a consequence of increased fluorescence intensity in cells with diminished Hsp104 function. When a similar mating was performed but with Sup35-GFP expressed in the [*PSI^+^*] partner, the fluorescence intensity of foci was Hsp104 independent (Figure 2C), but again, foci only remained detectable in microcolonies with inactive Hsp104 (Figure S3). Thus, Hsp104 appears to stimulate turnover of existing Sup35-GFP^[*PSI+*]^ complexes.

**Figure 5:**
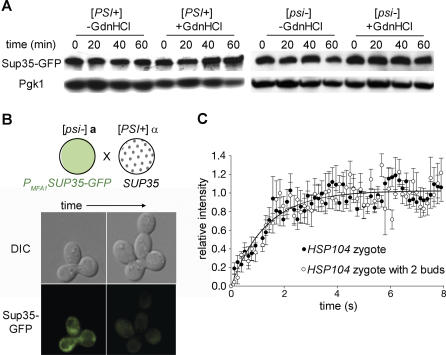
Loss of Sup35-GFP^[*PSI+*]^ Fluorescence in Wild-Type Cells Corresponds to Redistribution of Existing Fusion Protein to New Prion Complexes (A) The metabolic stability of Sup35-GFP was analyzed in [*PSI^+^*] (SY81) or [*psi^−^*] (SY87) yeast lysates upon inhibition of new protein synthesis with cycloheximide. An anti-Sup35 immunoblot is shown along with an anti-Pgk1 immunoblot of the same membrane as a control for loading. (B) Shown are representative images (*n* = 7) of a wild-type zygote (SY360 × SY581) constitutively expressing untagged *SUP35* from its endogenous locus (left image) and of the same zygote following the formation of a second daughter and first granddaughter (right image). The Sup35-GFP in these cells is that existing at the time of mating as described in Figure 2A. (C) FRAP recovery curves for a wild-type zygote (SY360 × SY581; black circles) and its daughter (white circles), as described in (B), are shown. The respective recovery times are 0.92 s (R^2^ = 0.85) and 0.95 s (R^2^ = 0.84).

What is the molecular basis of this loss in Sup35-GFP fluorescence? Hsp104-dependent degradation or remodeling (Figure 4D) of existing Sup35-GFP are two possibilities. To test the former idea, we monitored the metabolic stability of existing Sup35-GFP in [*PSI^+^*] and [*psi^−^*] cells in the presence or absence of Hsp104 function. Sup35-GFP accumulates to similar levels under all conditions at steady state as determined by Western blotting of lysates following SDS-PAGE (Figure 5A, time 0). These levels do not change over a 1-h time course following the addition of cycloheximide to inhibit new Sup35-GFP synthesis (Figure 5A). In contrast, the short-lived Matα2 protein is rapidly lost from yeast lysates following cycloheximide inhibition (Figure S4). These data together indicate that Sup35-GFP is stable and that its half-life is unaltered by Hsp104 activity.

Given these observations, we next considered the alternate explanation: Hsp104-dependent remodeling of Sup35-GFP^[*PSI+*]^ complexes. In this scenario, existing Sup35-GFP^[*PSI+*]^ complexes are fragmented into smaller pieces, which incorporate newly made, untagged Sup35 (Figure 4D). Although the initial and the remodeled complexes will be of similar size, their fluorescence intensities will be distinct since each contains a different ratio of GFP-fused and untagged Sup35 (Figure 4D). To test this idea, we monitored FRAP, a parameter influenced by diffusion rate and, therefore, particle size [49], in wild-type [*PSI^+^*] zygotes and in their daughters (Figure 5B and 5C). Sup35-GFP^[*PSI+*]^ fluorescence recovered with similar half-times in both cells (∼1 s), consistent with an Hsp104-dependent remodeling of existing prion complexes.

### Loss of Hsp104-Dependent Remodeling Limits Both Sup35-GFP^[*PSI+*]^ Transmission to Daughter Cells and the Subsequent Incorporation of Newly Synthesized Prion Protein

In addition to persistence of Sup35-GFP^[*PSI+*]^ foci in Hsp104-inactivated cells, we also noted a segregation bias (Figure 4B and 4C). Occasionally, the originating zygote could be identified in the microcolony by its characteristic shape (Figure 4B, outline). In these instances, we observed a large number of foci retained in zygotes (Figure 4B, arrowhead) and immediately adjacent cells, with progressively fewer foci in cells at the periphery. Thus, the decreased mobility of Sup35-GFP^[*PSI+*]^ complexes in Hsp104-inactivated cells (Figure 3A) correlated with inefficient transfer of these complexes to daughters.

**Figure 6:**
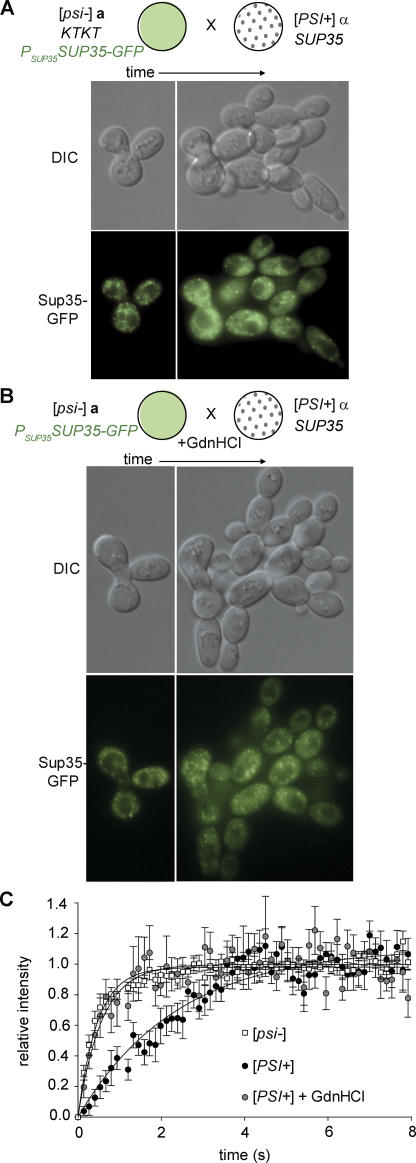
Newly Synthesized Sup35-GFP Is Inefficiently Incorporated into Limiting Prion Complexes Resulting from Hsp104 Inhibition and Cell Division (A) A *MAT**a*** [*psi^−^*] haploid with *KTKT* and *SUP35GFP* chromosomal replacements (SY751) was mated to a *MATα* [*PSI^+^*] haploid constitutively expressing *SUP35* (SY581) as shown in the diagram (top). Representative images of a zygote and microcolony derived from this zygote are shown (*n* = 7). (B) A *MAT**a*** [*psi^−^*] haploid with a *SUP35GFP* chromosomal replacement (SY87) was mated to a *MATα* [*PSI^+^*] haploid constitutively expressing *SUP35* (SY581) in the presence of GdnHCl as shown in the diagram. Representative images of a zygote and microcolony derived from this zygote are shown (*n* = 7). Under these conditions, the isolated zygote (left, [A] and [B]) continues to express Sup35-GFP, but lacks functional Hsp104. (C) FRAP recovery curves for [*psi^−^*] (SY360, white squares), [*PSI^+^*] (SY597, black circles) and [*PSI^+^*] cells after treatment with GdnHCl for 16–24 h (SY597, gray circles), which all constitutively express Sup35-GFP, are shown. The respective recovery times are: 0.33 s (R^2^ = 0.93), 1.16 s (R^2^ = 0.95), and 0.39 s (R^2^ = 0.79).

What consequence does biased segregation of existing Sup35-GFP^[*PSI+*]^ complexes have on conversion of newly made prion protein? To answer this question, we monitored Sup35-GFP fluorescence in Hsp104-inactivated [*psi^−^*] × [*PSI^+^*] zygotes and their daughters, which continued to synthesize the fusion. In contrast to microcolonies that no longer express Sup35-GFP (Figure 4B and 4C), diffuse fluorescence was detected in Hsp104-deficient daughter cells constitutively synthesizing Sup35-GFP (Figure 6A and 6B). Consistent with this observation, a rapidly recovering pool of Sup35-GFP was detected by FRAP in cells constitutively expressing the fusion upon prolonged exposure to GdnHCl (∼0.3 s for 50% recovery; Figure 6C). Accumulation of soluble Sup35-GFP in Hsp104-deficient microcolonies starkly contrasts with the quantitative incorporation of soluble Sup35-GFP into Sup35^[*PSI+*]^ complexes in zygotes (Figure 2B), likely reflecting a difference in the number of existing prion complexes in the two conditions [37]. Thus, individual prion complexes must have a finite conversion capacity in the absence of Hsp104 function.

### Static Sup35-GFP^[*PSI+*]^ Complexes Remain Substrates for Hsp104-Dependent Remodeling

**Figure 7:**
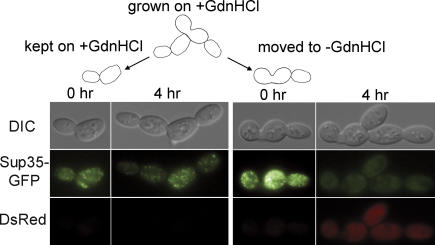
Sup35-GFP^[*PSI+*]^ Complexes, Which Persist after Hsp104 Inhibition, Remain Substrates for This Disaggregase upon Its Reactivation Wild-type zygotes (SY876 × 74D-694) constitutively expressing *SUP35* and *GST(UGA)DsRED* with existing Sup35-GFP (previously expressed from P*_MFA1_*) were isolated in the presence of GdnHCl and allowed to form microcolonies. As indicated in the schematic diagram, a portion of this colony was retained on medium containing GdnHCl (0 h, left), and the remainder of the colony was transferred to medium lacking GdnHCl (0 h, right). The cells were imaged again 4 h after the transfer. Representative DIC, GFP, and DsRed images are shown (*n* = 9).

Prion loss or curing by inactivation of Hsp104 is a reversible process provided that Hsp104 is reactivated in cells containing a limiting threshold of propagons [36]. How is efficient prion propagation restored to these cells? Based on our observations above, remodeling of existing static Sup35^[*PSI+*]^ complexes upon Hsp104 reactivation is one possibility. To test this idea, we isolated zygotes resulting from a cross between [*psi^−^*] cells expressing Sup35-GFP from P*_MFA1_* and unmarked [*PSI^+^*] cells on solid medium containing GdnHCl. Under these conditions, the zygotes do not continue to express the fusion [43]. These zygotes were then allowed to form microcolonies in the presence of GdnHCl, which were subsequently divided into two parts. One portion was retained on GdnHCl-containing medium (Figure 7, left), whereas the other was transferred to medium lacking GdnHCl (Figure 7, right). Fluorescent foci persisted in cells immediately after transfer to medium containing or lacking GdnHCl (Figure 7, time 0). Although continued treatment with GdnHCl preserved the integrity of existing complexes even in cells containing small numbers of foci (Figure 7, left), existing Sup35-GFP foci quickly resolved in cells transferred to medium lacking GdnHCl (Figure 7, right). Thus, immobile aggregates, which accumulate in GdnHCl-treated [*PSI^+^*] cells, remain substrates for Hsp104-dependent remodeling.

Is the resolution of these static Sup35-GFP^[*PSI+*]^ complexes upon Hsp104 reactivation relevant for the [*PSI^+^*] prion cycle? To test this idea, we also monitored the phenotype of the same cells upon removal of GdnHCl from the medium by assaying the nonsense codon readthrough-dependent expression of DsRed. Remodeling of existing Sup35-GFP foci correlated with a phenotypic switch from accurate translation termination (−DsRed) to the readthrough of stop codons (+DsRed; Figure 7), indicating the rapid inactivation of excess soluble Sup35. Thus, static Sup35-GFP^[*PSI+*]^ complexes preserve their capacity to direct the efficient incorporation of soluble Sup35^[*psi−*]^, but only when Hsp104 function is restored.

### Prion Complexes Become Mildly Toxic upon Loss of Hsp104 Function

Given their relative immobility, Sup35^[*PSI+*]^ complexes present in Hsp104-deficient cells may have detrimental effects on the organism. To test this idea, we first monitored the subcellular localization of a fusion between the stress-responsive transcription factor Msn2 and GFP. Msn2-GFP resides in the cytoplasm in unstressed cells, but in response to stress stimuli, it translocates into the nucleus where it induces expression of genes containing stress-response elements in their promoters [50]. In contrast to ethanol-treated cultures, [*PSI^+^*] and [*psi^−^*] cultures treated with GdnHCl retained Msn2-GFP in the cytoplasm (Figure S5), indicating that inactivation of Hsp104 did not produce an Msn2-mediated response in vivo.

**Figure 8:**
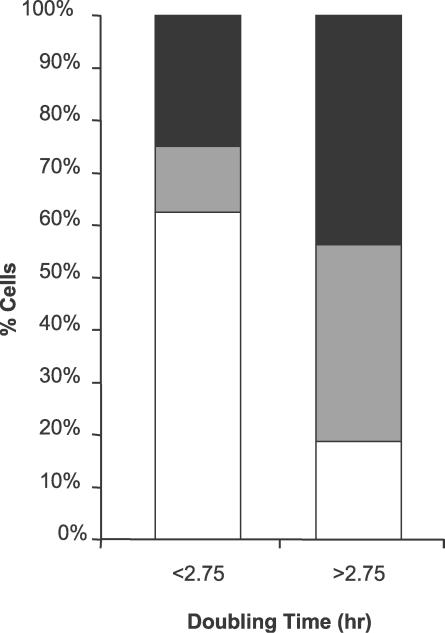
Propagon Numbers Correlate with Single-Cell Doubling Times Wild-type [*PSI^+^*] haploids (5V-H19) were analyzed for single-cell doubling time in the presence of GdnHCl and propagon numbers. The percentage of cells containing more than 1,000 (black), between 1,000 and 500 (gray), and fewer than 500 (white) propagons is indicated.

Although GdnHCl treatment does not activate a stress response in [*PSI^+^*] cultures (Figure S5), it does alter the cell-division cycle. In the presence of GdnHCl, [*PSI^+^*] cells produce their first daughter more slowly than untreated cells, whereas GdnHCl treatment had no effect on the division time of [*psi^−^*] cells (Table 1). This doubling-time extension could arise either from an interaction of GdnHCl with the prion phenotype or with prion complexes. Nonsense suppression, however, is not the underlying cause of this effect since expression of a Sup35 deletion mutant (ΔNM), which restores accurate termination without interfering with prion propagation by full-length Sup35 [51], does not reverse the doubling-time extension (3.7 ± 1.1 h). In contrast, we noted a correlation between doubling time and propagon number: cells with shorter doubling times (<2.75 h) tended to have fewer propagons than those with longer doubling times (Figure 8). Consistent with this idea, the [*PSI^+^*]- and GdnHCl-dependent doubling-time extension was most severe for formation of first daughters, as subsequent daughters were produced at more similar rates in the presence and absence of GdnHCl (Table 1). Thus, cell division, and perhaps the corresponding small decrease in the number of prion complexes (Figure 4B and 4C), at least partially alleviates the detrimental effect of inhibiting Hsp104 in [*PSI^+^*] cells.

**Table 1:**
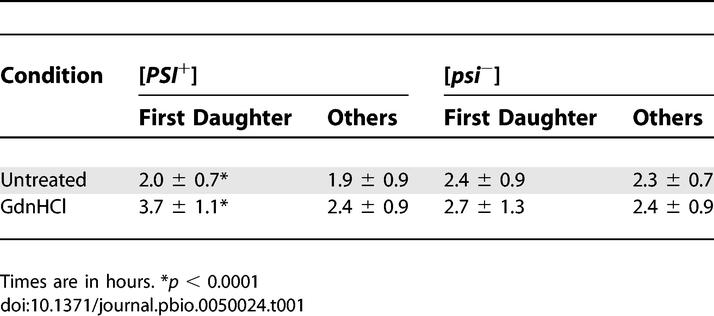
Single-Cell Doubling Times

## Discussion

Together, our in vivo studies probe the molecular mechanism by which Hsp104 mediates propagation of the Sup35/[*PSI^+^*] prion. We suggest that Hsp104 acts at a single point in the prion cycle to remodel existing Sup35^[*PSI+*]^ complexes (Figure 1). By tightly regulating expression of Sup35-GFP, we provide direct evidence for this event: the decreased fluorescence of existing Sup35-GFP^[*PSI+*]^ in wild-type microcolonies (Figure 4). Despite this change in fluorescence, the protein persists (Figure 5A) and remains assembled in complexes (Figure 5C). Because Sup35-GFP^[*PSI+*]^ foci remain uniformly detectable in wild-type [*PSI^+^*] cells that constitutively express the fusion [43], we interpret our observations as a redistribution of existing Sup35-GFP molecules to new complexes containing an excess of untagged Sup35 (Figure 4D), providing direct experimental support in vivo for fragmentation [6,35].

In the absence of Hsp104-dependent remodeling of Sup35-GFP^[*PSI+*]^ complexes, a number of crucial events in the [*PSI^+^*] prion cycle are compromised. First, Sup35-GFP^[*PSI+*]^ complexes increase in size, initially detected as brighter Sup35-GFP fluorescence (Figure 2B). By separately expressing Sup35-GFP in either [*PSI^+^*] or [*psi^−^*] cells and inhibiting fusion protein synthesis upon mating, we unambiguously link this change to incorporation of soluble Sup35-GFP^[*psi−*]^ into existing complexes (Figure 2). Importantly, when [*PSI^+^*] complexes are marked with Sup35-GFP prior to mating, no change in fluorescence intensity is detected upon Hsp104 inhibition, indicating that existing complexes do not laterally associate to form larger particles (Figure 2C). Thus, the number of Sup35-GFP^[*PSI+*]^ complexes becomes static when Hsp104 activity is lost, as previously predicted in mathematical models of curing [36,37].

The increase in fluorescence intensity of Sup35-GFP^[*PSI+*]^ complexes also correlates with a decrease in their mobility (Figure 3). Although this relative immobility is immediately apparent upon zygote formation when a large bolus of soluble Sup35 incorporates (Figure 3), the decreased mobility arises over time in GdnHCl-treated haploids as newly synthesized Sup35 continues to join complexes (Video S1 and unpublished data). In either case, this immobility provides a possible molecular explanation for the diminished transmission of propagons to daughter cells (Figure 4) [37,39] and the consequential decrease in the efficiency of incorporation of soluble Sup35^[*psi−*]^ (Figure 6) [32].

Can our observations of Sup35-GFP^[*PSI+*]^ dynamics in vivo explain the behavior of heritable prion particles? Several lines of evidence suggest that Sup35-GFP^[*PSI+*]^ foci are a robust proxy for propagons. First, Sup35-GFP is fully functional in translation termination, accumulates in cytoplasmic foci only in [*PSI^+^*] cells, supports [*PSI^+^*] propagation as the only copy of Sup35, and is regulated by Hsp104 levels in a manner that is indistinguishable from wild-type Sup35 [43]. Second, mathematical models of propagon dynamics upon Hsp104 inhibition predict a block in replication of these heritable units and their subsequent dilution by cell division [36]. The changes in Sup35-GFP foci that we observed upon Hsp104 inhibition (Figures 2–4 and 7) provide direct experimental support at the single-cell level for these predictions: Sup35-GFP^[*PSI+*]^ foci become static upon Hsp104 inhibition, and their reduced mobility correlates with an inefficient transfer to daughter cells (Figures 3 and 4). Third, genetic analyses of propagon transmission revealed a segregation bias in which mothers are more likely to retain prion seeds than their daughters [37]. The preferential accumulation of existing Sup35-GFP^[*PSI+*]^ in zygotes and their immediate progeny mirrors propagon behavior upon division (Figure 4). Fourth, propagon numbers increase independently of cell division and protein synthesis upon reactivation of Hsp104 [32,37]. In line with this observation, static Sup35-GFP^[*PSI+*]^ complexes are rapidly resolved coincidently with the bulk inactivation of soluble Sup35 upon removal of GdnHCl from the medium (Figure 7). The close alignment of our observations with the previous genetic characterizations of heritable prion units suggests that propagons are equivalent to or derived from Sup35-GFP^[*PSI+*]^ foci. If this relationship is indeed direct, counting propagons by dispersing colonies formed in the presence of GdnHCl is likely to yield an underestimate given the clear segregation bias that we observe for Sup35-GFP^[*PSI+*]^ foci (Figure 4), as previously suggested [37].

Our work also resolves some of the seemingly conflicting data present in the literature, which necessitated formation of multiple models for the role of Hsp104 in prion propagation. One of these controversies is the fate of existing Sup35-GFP^[*PSI+*]^ upon Hsp104 inhibition. Ness et al. observe persistent aggregation of existing Sup35 in GdnHCl-treated [*PSI^+^*] cultures using a biochemical assay [32]. In contrast, Wu et al. report disassembly of Sup35-GFP^[*PSI+*]^ complexes upon Hsp104 inhibition with the same treatment, as assessed by the accumulation of soluble Sup35-GFP^[*PSI+*]^ using microscopic approaches [41]. A key consideration in the interpretation of these studies is the status of Sup35 expression. Wu et al. monitored the behavior of bulk Sup35-GFP under conditions of on-going synthesis, whereas Ness et al. focused their studies on the dynamics of existing Sup35 only [32,41]. By regulating expression of Sup35-GFP, we resolve these apparent conflicts. Accumulation of soluble Sup35-GFP upon Hsp104 inhibition only occurs with continued synthesis of the fusion (Figures 4 and 6). Thus, prion complexes are not destabilized upon Hsp104 loss, but rather become more stable (Figure 4) and less efficient at incorporating newly synthesized Sup35 (Figure 6). Nonetheless, these static complexes remain biologically active substrates that are rapidly remodeled to efficient templates upon Hsp104 reactivation (Figure 7).

Given these observations, [*psi^−^*] cells must only appear when existing Sup35^[*PSI+*]^ complexes fail to segregate during cell division, a prediction at odds with a previous study, which reported prion curing in arrested cultures [41]. However, recent studies by Byrne, Cox, and Tuite have uncovered a high degree of lethality in the strain employed by Wu et al. upon release from α-factor arrest (L. Byrne, B. S. Cox, and M. F. Tuite, personal communication). Thus, the reported curing actually occurred in dividing cultures, but this division was overlooked due to the extensive, and counterbalancing, cell death.

Another point of controversy is the role of Hsp104 in the efficiency of Sup35^[*psi−*]^ conversion to the prion state. We observe quantitative inactivation of Sup35^[*psi−*]^ upon introduction of Sup35^[*PSI+*]^ in the absence of Hsp104 function in some cells, as monitored by the appearance of a translation termination defect (Figure 2B) [43], suggesting that Hsp104 is not directly required for efficient incorporation. However, the percentage of cells displaying the prion phenotype is diminished in Hsp104-inactivated zygotes. This observation is consistent with a primary role for Hsp104 in the generation of additional prion templates [6,35,36]. Specifically, the converting capacity of some cells falls below the threshold needed to inactivate the vast majority of soluble Sup35^[*psi−*]^ contributed by the [*psi^−^*] mating partner when Hsp104 is inhibited. Since Hsp104 is not available to generate additional prion seeds, this converting potential is dictated by the number of propagons present in the cell at the time of Hsp104 inhibition, a factor that varies widely in individual cells of a homogenous culture [37]. Consistent with this interpretation, newly synthesized Sup35-GFP accumulates in a soluble form soon after Hsp104 inhibition (Figure 6) [32], coincident with the loss of Sup35-GFP^[*PSI+*]^ remodeling (Figure 4), and this soluble pool is rapidly inactivated upon restoration of Hsp104 function and remodeling of existing Sup35-GFP^[*PSI+*]^ complexes (Figure 7).

An exclusive role for Hsp104 in the generation of new prion seeds is also compatible with observations in vitro. Sup35 self-assembly into amyloid fibers is accelerated by the addition of Hsp104 [13,33], and this effect has been linked to a steady-state increase in the abundance of an amyloidogenic intermediate [33,34]. One interpretation of this observation is that Hsp104 promotes the de novo formation of this species [33], but an alternate explanation is the Hsp104-dependent fragmentation of spontaneously arising intermediates. The incorporation of soluble protein into the smaller complexes predicted in this alternative scenario would also result in an apparent steady-state increase in the abundance of this form. Consistent with this interpretation, Hsp104 preferentially interacts with low molecular weight oligomers of Sup35 [52], which are intermediates in fiber assembly and disassembly reactions in vitro [53], and specifically alters the biophysical properties of Sup35-GFP^[*PSI+*]^ complexes in vivo (Figure 3). Thus, the enhanced conversion efficiency in vivo and in vitro is likely to be a secondary consequence of prion-complex remodeling by Hsp104 (Figure 4) [32].

Loss of Hsp104-dependent remodeling also uncovers biological consequences for single-cell differences in a superficially homogenous culture. In addition to the variation in translation termination efficiency, [*PSI* ^+^] cells lacking Hsp104 activity display a broader range of doubling times than their untreated counterparts (Table 1). Again, these distinctions likely arise from cell-to-cell variation in the number of prion particles [37] at the time of Hsp104 inhibition, with cells containing a larger number of prion particles being the most efficient at converting soluble Sup35, but also the most detrimental to cellular physiology (Figure 8).

How is remodeling mechanistically accomplished? Hsp104 forms a hexamer with a central pore [26], and previous studies suggest that Hsp104 functions by threading substrates through this channel [54,55]. How, then, might this extrusion lead to Sup35^[*PSI+*]^ remodeling and production of new prion complexes? Other members of the Clp/Hsp100 family may provide some insight. For example, ClpX extracts a single MuA monomer from a DNA-bound tetramer, an event that destabilizes the remainder of the complex and promotes dissociation [56–58]. Hsp104 could act similarly on Sup35^[*PSI+*]^ complexes by unfolding constituent monomers and thereby fragmenting the now destabilized oligomer. A future challenge, then, is to determine the contributions of each of these species, the unfolded monomer and the fragmented oligomer, to prion propagation and the viability of [*PSI* ^+^] cells.

## Materials and Methods

### Plasmid construction.

pRS306P_SUP35_SUP35-GFP, pRS303P_MFA1_ SUP35-GFP, pRS305P_LEXAop-GAL1_DsRED, pRS306P_GPD_LEXA-GAL4, and pRS304GST(UGA)DsRED were previously described [43]. For pRS304P_GPD_GSTDsRED, P*_GPD_* and the *CYC1* terminator were subcloned as a SacI-KpnI fragment from p424GPD [59] into pRS304 to create pRS304P_GPD_. Gluathione-S-transferase (GST) was PCR amplified with BamHI and EcoRI linkers, and DsRED with EcoRI and ClaI linkers, and these two fragments were subcloned into pRS304P_GPD_. Primer sequences are available upon request. The pRS306P_HSE_HSP104K218TK620T plasmid was kindly provided by S. Lindquist (Whitehead Institute, Massachusetts Institute of Technology). The Msn2-GFP expression plasmid (from P*_ADH1_* in YCplac111) was a kind gift from C. Schueller (Max F Perutz Laboratories, University and BioCenter Vienna).

### Strain construction.

Strains used in this study are listed in Table 2 and are derivatives of 74D-694 [20] unless otherwise indicated. *KTKT* substitutions were constructed by two-step replacement at the *HSP104* locus using EagI-digested pRS306P_HSE_HSP104K218TK620T and verified by [*PSI^+^*] loss and sequencing. SUP35-GFP substitutions were constructed by two-step replacement as previously described [43]. Plasmids were integrated at the indicated locus by linearizing the plasmid with the indicated restriction endonuclease within the nutritional marker: pRS304P_GPD_GST(UGA)DsRED (TRP1, EcoRV), pRS303P_MFA1_SUP35-GFP (HIS3, Eco47III), and pRS304P_GPD_GSTDsRED (TRP1, EcoRV).

**Table 2:**
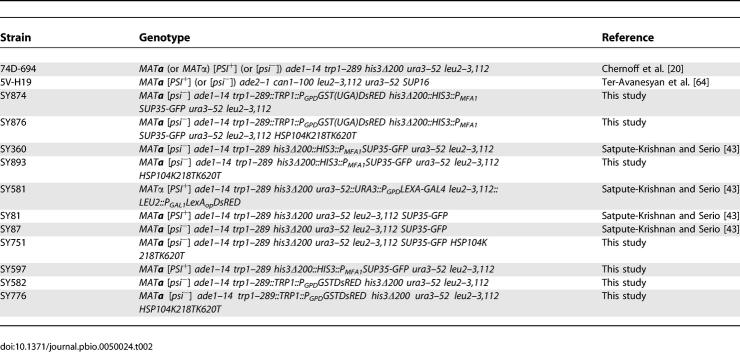
Yeast Strains

### Zygote isolation.

Matings and zygote isolation and confirmation were performed as previously described [43]. Where indicated, mating cells were treated with 3 mM GdnHCl for at least 2.5 h before zygote isolation.

### Imaging and digital processing.

Cells were observed using a Zeiss Axioplan 2 (Carl Zeiss, MicroImaging,Oberkochen, Germany) equipped with a 100× αPlan-FLUAR objective and Hamamatsu-ORCA camera (Hamamatsu Photonics, Hamamatsu City, Japan) as previously described [43].

### FRAP.

FRAP experiments were performed on a Leica TCS SP2 AOBS spectral confocal microscope equipped with an 100× oil objective (NA 1.4) and Argon/Helium Neon laser, and data was captured using the LCS 2.5 software (Leica Microsystems, Wetzlar, Germany). Frames (16× zoom, 256 × 100 pixels, 2 Airy units) were collected at 0.13 s bidirectionally at 1,000 Hz and 488-nm excitation using GFP conditions (500 nm to 560 nm). Laser intensity was set to 3%–4% for collection and 100% for bleaching. The FRAP region of interest (ROI) was 0.5 μm in diameter and was drawn in the cytoplasm to encompass soluble Sup35-GFP or foci and to avoid the vacuole. Software-generated intensity values for the ROI represented the mean across all pixels. FRAP data were independently corrected for photobleaching (by comparison with a neighboring region in the same cell) and normalized against prebleach values as previously described [60].

### Protein analysis.

The metabolic stabilities of Sup35-GFP and Matα2 were analyzed by the post-alkaline extraction protocol [61] followed by SDS-PAGE and immunoblotting with anti-Pgk1 (Molecular Probes, Eugene, Oregon, United States) and either anti-Matα2 or anti-Sup35 antiserum as previously described [43,62]. Yeast cultures were treated with 200-μg/ml cycloheximide at time 0 prior to lysis as previously described [63].

### Doubling-time analysis.

Large-budded 5V-H19 cells were micromanipulated from exponentially growing liquid YPD cultures onto solid YPD either containing or lacking 5 mM GdnHCl. Buds were removed and discarded at the earliest possible point (typically within the first hour). Mothers were then monitored over time, and the point at which the next daughter could be removed was recorded.

### Propagon counts.

Individual cells were micromanipulated onto YPD containing 5 mM GdnHCl and incubated at 30 °C for 7 d. Colonies were removed from the plate as previously described [37] and plated directly onto SD-ade. After incubation at 30 °C for 10 d, adenine prototrophs were scored. In control experiments, more than 90% of adenine prototrophs were GdnHCl curable.

## Supporting Information

Figure S1 Newly Synthesized Sup35-GFP Joins Existing Sup35 Complexes in the Absence of Hsp104 Function *MAT* ***a*** [*psi^−^*] *KTKT* haploids (SY751) were mated to *MATα* haploids constitutively expressing *SUP35-GFP* (SY581) as indicated in the schematic diagram (top). Zygotes from this cross were isolated (left); existing Sup35-GFP was then photobleached (middle), and the zygote was allowed to form a microcolony (right). Representative differential interference contrast (DIC) and GFP images are shown (*n* = 7).
(5.0 MB TIF)

Figure S2 Sup35-GFP^[*PSI+*]^ Complexes Become Immobile after Inhibition of Hsp104 FRAP time courses for Sup35-GFP^[*PSI+*]^ in wild-type zygotes (SY360 × SY581) in the presence of GdnHCl (white squares, *n* = 5) are shown. The black arrow marks the bleach point. Error bars represent standard error of the mean.
(86 KB TIF)

Figure S3 Sup35-GFP^[*PSI+*]^ Remains Detectable in Microcolonies Lacking Hsp104 Function *MAT* ***a*** [*PSI^+^*] P*_MFA1_ SUP35-GFP* haploids were mated to *MATα* [*psi^−^*] haploids constitutively expressing *SUP35* in the presence or absence of functional Hsp104. For each cross, a zygote (left) and a microcolony derived from that zygotes (right) are shown. Representative images from a wild-type (SY597 × SY582; left, *n* = 15), *KTKT* (SY597 × SY776; middle, *n* = 8), or a wild-type cross in the presence of GdnHCl (SY597 × SY582; right, *n* = 11) are shown.
(5.5 MB TIF)

Figure S4 Matα2 Is Short-Lived The metabolic stability of Matα2 was analyzed in *MATα* 74D-694 cells after the inhibition of new protein synthesis with cycloheximide. Lysates from *MAT* ***a*** 74D-694 cells, which do not express Matα2, are included as a negative control. An anti-Pgk1 blot of the same membrane is shown as a loading control.
(1.3 MB TIF)

Figure S5 The Stress-Responsive Transcription Factor Msn2 Is Not Activated in [*PSI^+^*] Cells Treated with GdnHCl Shown are representative DIC and GFP images of *MAT* ***a*** 74D-694 [*PSI^+^*] or *MAT**a*** 74D-694 [*psi^−^*] cells constitutively expressing an Msn2-GFP fusion protein from P*_ADH1_* in untreated, ethanol-treated (+EtOH), or GdnHCl-treated cultures.
(1.5 MB TIF)

Video S1 Sup35-GFP^[*PSI+*]^ Complexes Become Immobile in GdnHCl-Treated Haploids Shown is a 5-s video (1 frame/s) of a [*PSI^+^*] strain constitutively expressing Sup35-GFP (SY597) after a 24-h treatment with GdnHCl.
(8 KB MOV)

## Abbreviations

DIC: differential interference contrast
FRAP: fluorescence recovery after photobleaching
GFP: green fluorescent protein

## References

[pbio-0050024-b001] Griffith J (1967). Self-replication and scrapie. Nature.

[pbio-0050024-b002] Prusiner SB (1982). Novel proteinaceous infectious particles cause scrapie. Science.

[pbio-0050024-b003] Wickner RB (1994). [URE3] as an altered URE2 protein: Evidence for a prion analog in Saccharomyces cerevisiae. Science.

[pbio-0050024-b004] Masison DC, Wickner RB (1995). Prion-inducing domain of yeast Ure2p and protease resistance of Ure2p in prion-containing cells. Science.

[pbio-0050024-b005] Patino MM, Liu JJ, Glover JR, Lindquist S (1996). Support for the prion hypothesis for inheritance of a phenotypic trait in yeast. Science.

[pbio-0050024-b006] Paushkin SV, Kushnirov VV, Smirnov VN, Ter-Avanesyan MD (1996). Propagation of the yeast prion-like [PSI^+^] determinant is mediated by oligomerization of the SUP35-encoded polypeptide chain release factor. EMBO J.

[pbio-0050024-b007] Brachmann A, Baxa U, Wickner RB (2005). Prion generation in vitro: Amyloid of Ure2p is infectious. EMBO J.

[pbio-0050024-b008] King CY, Diaz-Avalos R (2004). Protein-only transmission of three yeast prion strains. Nature.

[pbio-0050024-b009] Tanaka M, Chien P, Naber N, Cooke R, Weissman JS (2004). Conformational variations in an infectious protein determine prion strain differences. Nature.

[pbio-0050024-b010] Glover JR, Kowal AS, Schirmer EC, Patino MM, Liu JJ (1997). Self-seeded fibers formed by Sup35, the protein determinant of [PSI^+^], a heritable prion-like factor of S. cerevisiae. Cell.

[pbio-0050024-b011] King CY, Tittmann P, Gross H, Gebert R, Aebi M (1997). Prion-inducing domain 2–114 of yeast Sup35 protein transforms in vitro into amyloid-like filaments. Proc Natl Acad Sci U S A.

[pbio-0050024-b012] Taylor KL, Cheng N, Williams RW, Steven AC, Wickner RB (1999). Prion domain initiation of amyloid formation in vitro from native Ure2p. Science.

[pbio-0050024-b013] Krzewska J, Melki R (2006). Molecular chaperones and the assembly of the prion Sup35p, an in vitro study. EMBO J.

[pbio-0050024-b014] Jones GW, Tuite MF (2005). Chaperoning prions: The cellular machinery for propagating an infectious protein?. Bioessays.

[pbio-0050024-b015] Moriyama H, Edskes HK, Wickner RB (2000). [URE3] prion propagation in Saccharomyces cerevisiae: Requirement for chaperone Hsp104 and curing by overexpressed chaperone Ydj1p. Mol Cell Biol.

[pbio-0050024-b016] Roberts BT, Moriyama H, Wickner RB (2004). [URE3] prion propagation is abolished by a mutation of the primary cytosolic Hsp70 of budding yeast. Yeast.

[pbio-0050024-b017] Schwimmer C, Masison DC (2002). Antagonistic interactions between yeast [PSI^+^] and [URE3] prions and curing of [URE3] by Hsp70 protein chaperone Ssa1p but not by Ssa2p. Mol Cell Biol.

[pbio-0050024-b018] Chernoff YO, Newnam GP, Kumar J, Allen K, Zink AD (1999). Evidence for a protein mutator in yeast: Role of the Hsp70-related chaperone ssb in formation, stability, and toxicity of the [PSI] prion. Mol Cell Biol.

[pbio-0050024-b019] Kryndushkin DS, Smirnov VN, Ter-Avanesyan MD, Kushnirov VV (2002). Increased expression of Hsp40 chaperones, transcriptional factors, and ribosomal protein Rpp0 can cure yeast prions. J Biol Chem.

[pbio-0050024-b020] Chernoff YO, Lindquist SL, Ono B, Inge-Vechtomov SG, Liebman SW (1995). Role of the chaperone protein Hsp104 in propagation of the yeast prion-like factor [PSI^+^]. Science.

[pbio-0050024-b021] Newman GP, Wegrzyn RD, Lindquist SL, Chernoff YO (1999). Antagonistic interactions between yeast chaperones Hsp104 and Hsp70 in prion curing. Mol Cell Biol.

[pbio-0050024-b022] Jung G, Jones G, Wegrzyn RD, Masison DC (2000). A role for cytosolic hsp70 in yeast [PSI^+^] prion propagation and [PSI^+^] as a cellular stress. Genetics.

[pbio-0050024-b023] Chacinska A, Szczesniak B, Kochneva-Pervukhova NV, Kushnirov VV, Ter-Avanesyan MD (2001). Ssb1 chaperone is a [PSI^+^] prion-curing factor. Curr Genet.

[pbio-0050024-b024] Kushnirov VV, Kryndushkin DS, Boguta M, Smirnov VN, Ter-Avanesyan MD (2000). Chaperones that cure yeast artificial [PSI^+^] and their prion-specific effects. Curr Biol.

[pbio-0050024-b025] Neuwald AF, Aravind L, Spouge JL, Koonin EV (1999). AAA+: A class of chaperone-like ATPases associated with the assembly, operation, and disassembly of protein complexes. Genome Res.

[pbio-0050024-b026] Parsell DA, Kowal AS, Lindquist S (1994). Saccharomyces cerevisiae Hsp104 protein. Purification and characterization of ATP-induced structural changes. J Biol Chem.

[pbio-0050024-b027] Parsell DA, Kowal AS, Singer MA, Lindquist S (1994). Protein disaggregation mediated by heat-shock protein Hsp104. Nature.

[pbio-0050024-b028] Sanchez Y, Lindquist SL (1990). HSP104 required for induced thermotolerance. Science.

[pbio-0050024-b029] Derkatch IL, Bradley ME, Zhou P, Chernoff YO, Liebman SW (1997). Genetic and environmental factors affecting the de novo appearance of the [PSI^+^] prion in Saccharomyces cerevisiae. Genetics.

[pbio-0050024-b030] Osherovich LZ, Weissman JS (2001). Multiple Gln/Asn-rich prion domains confer susceptibility to induction of the yeast [PSI^+^] prion. Cell.

[pbio-0050024-b031] Kimura Y, Koitabashi S, Kakizuka A, Fujita T (2004). The role of pre-existing aggregates in Hsp104-dependent polyglutamine aggregate formation and epigenetic change of yeast prions. Genes Cells.

[pbio-0050024-b032] Ness F, Ferreira P, Cox BS, Tuite MF (2002). Guanidine hydrochloride inhibits the generation of prion “seeds” but not prion protein aggregation in yeast. Mol Cell Biol.

[pbio-0050024-b033] Shorter J, Lindquist S (2004). Hsp104 catalyzes formation and elimination of self-replicating Sup35 prion conformers. Science.

[pbio-0050024-b034] Shorter J, Lindquist S (2006). Destruction or potentiation of different prions catalyzed by similar Hsp104 remodeling activities. Mol Cell.

[pbio-0050024-b035] Kushnirov VV, Ter-Avanesyan MD (1998). Structure and replication of yeast prions. Cell.

[pbio-0050024-b036] Eaglestone SS, Ruddock LW, Cox BS, Tuite MF (2000). Guanidine hydrochloride blocks a critical step in the propagation of the prion-like determinant [PSI^+^] of Saccharomyces cerevisiae. Proc Natl Acad Sci U S A.

[pbio-0050024-b037] Cox B, Ness F, Tuite M (2003). Analysis of the generation and segregation of propagons: Entities that oropagate the [PSI^+^] prion in yeast. Genetics.

[pbio-0050024-b038] Kryndushkin DS, Alexandrov IM, Ter-Avanesyan MD, Kushnirov VV (2003). Yeast [PSI^+^] prion aggregates are formed by small Sup35 polymers fragmented by Hsp104. J Biol Chem.

[pbio-0050024-b039] Wegrzyn RD, Bapat K, Newnam GP, Zink AD, Chernoff YO (2001). Mechanism of prion loss after Hsp104 inactivation in yeast. Mol Cell Biol.

[pbio-0050024-b040] Kawai-Noma S, Ayano S, Pack CG, Kinjo M, Yoshida M (2006). Dynamics of yeast prion aggregates in single living cells. Genes Cells.

[pbio-0050024-b041] Wu YX, Greene LE, Masison DC, Eisenberg E (2005). Curing of yeast [PSI^+^] prion by guanidine inactivation of Hsp104 does not require cell division. Proc Natl Acad Sci U S A.

[pbio-0050024-b042] Cox B (1965). [PSI], a cytoplasmic suppressor of super-suppression in yeast. Heredity.

[pbio-0050024-b043] Satpute-Krishnan P, Serio TR (2005). Prion protein remodelling confers an immediate phenotypic switch. Nature.

[pbio-0050024-b044] Ferreira PC, Ness F, Edwards SR, Cox BS, Tuite MF (2001). The elimination of the yeast [PSI^+^] prion by guanidine hydrochloride is the result of Hsp104 inactivation. Mol Microbiol.

[pbio-0050024-b045] Grimminger V, Richter K, Imhof A, Buchner J, Walter S (2004). The prion curing agent guanidinium chloride specifically inhibits ATP hydrolysis by Hsp104. J Biol Chem.

[pbio-0050024-b046] Jung G, Masison DC (2001). Guanidine hydrochloride inhibits Hsp104 activity in vivo: A possible explanation for its effect in curing yeast prions. Curr Microbiol.

[pbio-0050024-b047] Zhouravleva G, Frolova L, Le Goff X, Le Guellec R, Inge-Vechtomov S (1995). Termination of translation in eukaryotes is governed by two interacting polypeptide chain release factors, eRF1 and eRF3. EMBO J.

[pbio-0050024-b048] Bradley ME, Bagriantsev S, Vishveshwara N, Liebman SW (2003). Guanidine reduces stop codon read-through caused by missense mutations in SUP35 or SUP45. Yeast.

[pbio-0050024-b049] Lippincott-Schwartz J, Altan-Bonnet N, Patterson GH (2003). Photobleaching and photoactivation: Following protein dynamics in living cells. Nat Cell Biol.

[pbio-0050024-b050] Gorner W, Durchschlag E, Martinez-Pastor MT, Estruch F, Ammerer G (1998). Nuclear localization of the C2H2 zinc finger protein Msn2p is regulated by stress and protein kinase A activity. Genes Dev.

[pbio-0050024-b051] Ter-Avanesyan MD, Dagkesamanskaya AR, Kushnirov VV, Smirnov VN (1994). The SUP35 omnipotent suppressor gene is involved in the maintenance of the non-Mendelian determinant [PSI^+^] in the yeast Saccharomyces cerevisiae. Genetics.

[pbio-0050024-b052] Narayanan S, Bosl B, Walter S, Reif B (2003). Importance of low-oligomeric-weight species for prion propagation in the yeast prion system Sup35/Hsp104. Proc Natl Acad Sci U S A.

[pbio-0050024-b053] Narayanan S, Walter S, Reif B (2006). Yeast prion-protein, Sup35, fibril formation proceeds by addition and substraction of oligomers. Chembiochem.

[pbio-0050024-b054] Lum R, Tkach JM, Vierling E, Glover JR (2004). Evidence for an unfolding/threading mechanism for protein disaggregation by Saccharomyces cerevisiae Hsp104. J Biol Chem.

[pbio-0050024-b055] Masison DC, Hung GC (2006). N-terminal domain of yeast Hsp104 chaperone is dispensable for thermotolerance and prion propagation but necessary for curing prions by Hsp104 overexpression. Genetics.

[pbio-0050024-b056] Levchenko I, Luo L, Baker TA (1995). Disassembly of the Mu transposase tetramer by the ClpX chaperone. Genes Dev.

[pbio-0050024-b057] Kruklitis R, Welty DJ, Nakai H (1996). ClpX protein of Escherichia coli activates bacteriophage Mu transposase in the strand transfer complex for initiation of Mu DNA synthesis. EMBO J.

[pbio-0050024-b058] Burton BM, Williams TL, Baker TA (2001). ClpX-mediated remodeling of Mu transpososomes: Selective unfolding of subunits destabilizes the entire complex. Mol Cell.

[pbio-0050024-b059] Mumberg D, Muller R, Funk M (1995). Yeast vectors for the controlled expression of heterologous proteins in different genetic backgrounds. Gene.

[pbio-0050024-b060] Axelrod D, Koppel DE, Schlessinger J, Elson E, Webb WW (1976). Mobility measurement by analysis of fluorescence photobleaching recovery kinetics. Biophys J.

[pbio-0050024-b061] Kushnirov VV (2000). Rapid and reliable protein extraction from yeast. Yeast.

[pbio-0050024-b062] Hochstrasser M, Varshavsky A (1990). In vivo degradation of a transcriptional regulator: The yeast alpha 2 repressor. Cell.

[pbio-0050024-b063] Laney JD, Hochstrasser M (2003). Ubiquitin-dependent degradation of the yeast Mat(alpha)2 repressor enables a switch in developmental state. Genes Dev.

[pbio-0050024-b064] Ter-Avanesyan MD, Kushnirov VV, Dagkesamanskaya AR, Didichenko SA, Chernoff YO (1993). Deletion analysis of the SUP35 gene of the yeast Saccharomyces cerevisiae reveals two non-overlapping functional regions in the encoded protein. Mol Microbiol.

